# Refractory Tunnel Infections Caused by Multidrug-Resistant Mycobacterium abscessus Requiring Peritoneal Dialysis Catheter Removal

**DOI:** 10.7759/cureus.104147

**Published:** 2026-02-23

**Authors:** Takahiro Morino, Kouji Shibuya, Teruki Shin, Tetsuya Noguchi

**Affiliations:** 1 Internal Medicine, Gojinkai Sumiyoshigawa Hospital, Kobe, JPN; 2 Surgery, Gojinkai Sumiyoshigawa Hospital, Kobe, JPN; 3 Internal Medicine, Rakuwakai Otowa Memorial Hospital, Kyoto, JPN; 4 Internal Medicine, Gojinkai Sumiyosigawa Hospital, Kobe, JPN

**Keywords:** catheter removal, multidrug resistance, mycobacterium abscessus (m. abscessus), peritoneal dialysis (pd), tunnel infections (ti)

## Abstract

Tunnel infections (TI) remain a challenging peritoneal dialysis (PD) complication, particularly when caused by *Mycobacterium abscessus* (*M. abscessus*). *M. abscessus* often exhibits multidrug resistance and frequently causes PD catheter removal. We report a case of TI due to multidrug-resistant* M. abscessus* that failed to respond to antimicrobial therapy and required catheter removal. A 58‑year‑old man on PD for end‑stage renal disease (ESRD) presented with localized abdominal tenderness and erythema with purulent discharge along the PD catheter tunnel tract. Levofloxacin (LVFX) was initiated empirically. *M. abscessus* was isolated on day 4, and susceptibility testing on day 12 revealed multidrug resistance to antimicrobial therapy, including antituberculosis agents. Despite continued antimicrobial therapy, the infection persisted, and the catheter was removed on day 14. The patient subsequently transitioned to hemodialysis and recovered uneventfully. An optimal therapeutic strategy for TI has not yet been clearly established in current clinical guidelines. In this case, the removal of the PD catheter two days after confirmation of multidrug resistance likely contributed to treatment success. In case of *M. abscessus*-related TI, prompt catheter removal should be considered as one of the treatment options.

## Introduction

Peritoneal dialysis (PD) is one of the renal replacement therapies (RRT) for patients with end-stage renal disease (ESRD), but infectious complications remain major barriers to long-term continuation of PD. Among these, PD catheter-related infections can progress to the point of necessitating catheter removal and, in severe cases, may lead to refractory peritonitis with potentially fatal outcomes. Thus, reversible preventive strategies and prompt intervention are critically important.

In recent years, an increasing number of reports have identified non-tuberculous mycobacteria (NTM) as causative bacteria of such infections [1]. Particularly noteworthy among NTM are the rapidly growing mycobacteria (RGM), which are characterized by visible colony formation within one week of culture [1]. *Mycobacterium abscessus* (*M. abscessus*), classified as RGM [2], is one of the pathogens associated with catheter-related infections. It is capable of forming biofilms in vitro that result in an increase of multidrug resistance to antimicrobial therapies [3], contributing to a refractory clinical course and potentially requiring PD catheter removal or even discontinuation of PD therapy [4].

Despite the clinical significance of these infections, the optimal treatment strategy for tunnel infections (TI) remains undefined in the most recent 2023 revision of the International Society for Peritoneal Dialysis (ISPD) guidelines [5]. Here, we report a case in which prompt removal of a PD catheter led to successful treatment of TI caused by multidrug-resistant *M. abscessus*.

## Case presentation

A 58‑year‑old man with ESRD secondary to diabetic kidney disease (DKD) had started PD two years ago. The patient was obese with a body mass index (BMI) of 33.8 and had frequent bathing habits, so the exit site for the PD catheter was created in the upper abdominal exit. Hemodialysis was added six months earlier due to insufficient fluid control.

Four months before presentation, erythema and purulent discharge were observed at the PD catheter exit site, raising suspicion for exit site infections (ESI). Culture of the purulent discharge identified coagulase-negative staphylococci (CNS). Daily topical mupirocin was applied to the exit site, but the infection did not resolve. Subcutaneous pathway diversion was performed, relocating the exit site to the lower abdomen. The infection resolved after the procedure.

**Figure 1:**
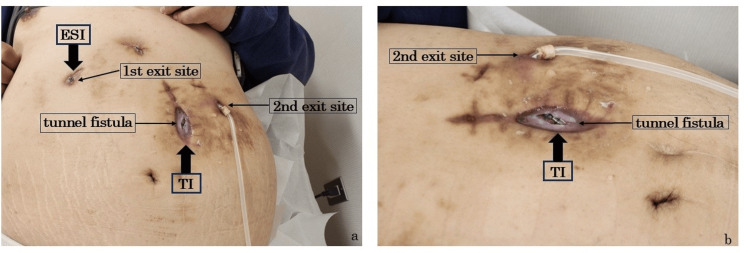
Appearance of exit site and tunnel fistula in the patient's abdomen a: distant view; b: close-up view ESI: exit site infections; TI: tunnel infections This figure shows erythema and discharge along a subcutaneous tunnel fistula. ESI occurred at the 1st exit site, and TI occurred at the tunnel fistula. The patient was obese and had frequent bathing habits, so the 1st exit site for the PD catheter was created in the upper abdomen. After ESI occurred, subcutaneous pathway diversion was performed, relocating the 2nd exit site to the lower abdomen.

Several days prior to the current presentation, the patient noted localized abdominal tenderness. Erythema and purulent discharge were observed at the tunnel fistula of the PD catheter, prompting a visit to our hospital. At the time of the outpatient visit, his vital signs were stable. The abdomen was soft and non-distended. Localized tenderness was noted in the left lateral abdomen, but peritoneal signs were not present. Bowel sounds were normal. The PD exit site showed erythema, and the tunnel fistula exhibited both erythema and exudate, with purulent discharge expressible upon compression (Figure 1).

Laboratory tests revealed a white blood cell (WBC) count of 7,500/μL (normal range: 4,000-8,000) and a C-reactive protein (CRP) level of 0.8 mg/dL (normal level: ≤0.4), indicating no significant systemic inflammatory response. Peritoneal fluid analysis showed a WBC count of 83/μL. A count of fewer than 100/μL without neutrophil predominance is generally considered not suggestive of peritonitis.

Although an ultrasound examination was not performed, the presence of redness, tenderness, and purulent discharge at the tunnel fistula indicated clinical signs of inflammation, leading to a diagnosis of TI. Levofloxacin (LVFX) therapy was initiated empirically, but on day 4 of illness, *M. abscessus* was isolated from a culture obtained from the tunnel fistula. As antimicrobial susceptibility results were pending, LVFX was continued. However, purulent discharge from the tunnel fistula persisted. On day 12, susceptibility testing revealed that the M. abscessus strain was multidrug resistant to antimicrobial therapies, including resistance to antituberculous agents (Table 1). Conservative treatment was deemed ineffective.

**Table 1:** Antimicrobial susceptibility testing of M. abscessus MIC: minimum inhibitory concentration; R: resistant Susceptibility interpretation was performed according to the "Performance standards for antimicrobial susceptibility testing," 22nd informational supplement; Clinical and Laboratory Standards Institute (CLSI) document M100-S22 (Wayne, PA: CLSI, 2012).

Antimicrobial	MIC (μg/mL)	Interpretation
Rifampicin (RFP)	>32	R
Streptomycin (SM)	128	R
Kanamycin (KM)	32	R
Ethambutol (EB)	64	R
Levofloxacin (LVFX)	>32	R
Amikacin (AMK)	>16	R

**Figure 2:**
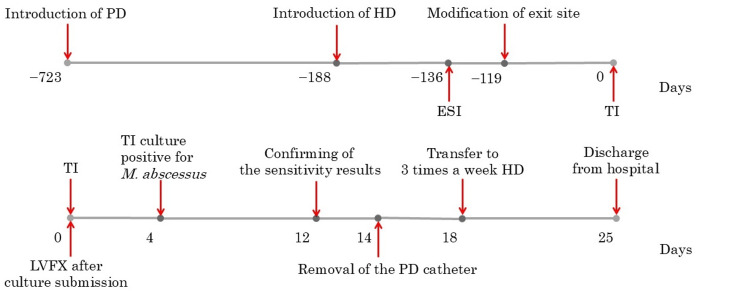
Timeline of the course of events PD: peritoneal dialysis; HD: hemodialysis; ESI: exit site infections; TI: tunnel infections; LVFX: levofloxacin; *M. abscessus*: *Mycobacterium abscessus* PD had been initiated two years before, but combined therapy with hemodialysis was introduced for insufficient fluid control six months before. Four months before, subcutaneous pathway diversion was performed after ESI. After diagnosis of TI LVFX was initiated empirically. On day 4 of illness, *M. abscessus* was isolated from a culture specimen. On day 12, susceptibility testing revealed multidrug resistance to antimicrobial therapies, including resistance to antituberculosis agents. The patient was hospitalized on day 13, and surgical catheter removal was performed on day 14. He transitioned to thrice-weekly hemodialysis on day 18 and was discharged in stable condition on day 25.

The patient was hospitalized on day 13, and the PD catheter was surgically removed on day 14. Hemodialysis three times weekly was initiated on day 18. The postoperative course was uneventful, and the patient was discharged on day 25. The complete timeline of the course of events is depicted in Figure 2.

## Discussion

Infections related to PD have been identified as the most important clinical concern for patients, caregivers, and clinicians participating in the Standardised Outcomes in Nephrology-Peritoneal Dialysis (SONG-PD) initiative [6]. Among these, catheter-related infections, which are defined as pathogen infections at the outer periphery of the tissue passage section of the PD catheter, are classified into ESI/TI [5].

According to the revised 2023 ISPD guidelines, ESIs are defined only when they show the presence of purulent discharge, regardless of whether or not clinical signs of inflammation, such as erythema, tenderness, swelling, granulation tissue, or crusting are observed [5]. On the other hand, TI is defined as the presence of clinical signs of inflammation (including erythema, swelling, tenderness, or induration) along the subcutaneous tunnel tract of the catheter, regardless of whether ultrasonographic imaging shows fluid collection along the tunnel [5].

This case exhibited erythema at the exit site and demonstrated clinical signs of inflammation, but lacked purulent discharge, making a definitive diagnosis of ESI insufficiently supported. Although ultrasonographic assessment was not performed, erythema, tenderness, and purulent discharge were shown at the tunnel fistula, so we diagnosed this case as TI by indicating the presence of clinical signs consistent with them.

This case involved a TI caused by *M. abscessus*. *M. abscessus* is known to exhibit multidrug resistance to macrolide antimicrobials such as clarithromycin (CAM) and erythromycin (EM) by inducing resistance through the erm (41) gene [7], and to form biofilm-like microcolonies on medical devices such as PD catheters [3]. *M. abscessus* is classified as RGM among NTM, forming visible colonies within one week of culture initiation [2]. In recent years, RGM have been increasingly reported as causative bacteria in catheter-related infections. Although the reasons for this trend are unknown, RGM are considered environmental bacteria commonly found in soil and water systems. Previous reports have indicated that infections caused by RGM occur more frequently in immunocompromised patients [1].

It is well-established that both diabetic and dialysis patients show impaired cellular immunity, which plays a central role in host defense against mycobacterial, viral, and fungal infections [8,9]. This case, in which the patient had initiated PD due to DKD, is presumed to be at high risk for impaired cellular immunity, so it was considered to be one of the factors contributing to the *M. abscessus* infection.

Additionally, this patient reported bathing approximately every other day but did so without using protective stoma dressings. Previous prospective cohort studies have demonstrated that the use of stoma dressings during daily showering significantly reduces the incidence of ESI [10]. Therefore, it could not be ruled out that the absence of such protective measures in this case may have contributed to the development of the infection.

The revised 2022 ISPD peritonitis guidelines propose that peritonitis caused by NTM should be treated with both appropriate antimicrobial therapy and PD catheter removal [11]. However, the updated 2023 ISPD guidelines on catheter-related infections do not clearly define standardized treatment protocols for ESI/TI. In cases of NTM-related ESI, it is recommended to administer at least a four-month course of two antimicrobial agents with documented in vitro activity against the clinically isolated bacteria. For refractory cases, PD catheter removal, simultaneous catheter removal and reinsertion (SCRR), cuff shaving or excision, or relocation of the exit site should be considered [5].

Regarding TI, at least a three-week course of effective antimicrobial agents, based on culture results and in vitro susceptibility testing, should be administered. If clinical improvement is achieved, administration should be continued for the recommended duration. If the infection is resistant to antimicrobial therapy, catheter removal or SCRR is recommended [5].

A review of the literature revealed 11 reported cases, including this case, of TI caused by *M. abscessus*, when considering cases with concomitant ESI [12-20] (Table 2). Among these, PD catheter removal was performed in nine cases, of which two involved SCRR. However, there have also been two reported cases in which PD could be continued without interruption.

**Table 2:** Reported M. abscessus TI in patients with PD catheters F: female; M: male; PD: peritoneal dialysis; HD: hemodialysis; ESI: exit site infections; TI: tunnel infections; SCRR: simultaneous catheter removal and reinsertion

Reference	Age	Sex	Index infection	Primary intervention	Outcome
Tsai SF (2013) [14]	50	M	ESI/TI	Antimicrobials	PD continued
Jo et al. (2012) [16]	69	M	ESI/TI	Removal of catheter	Switch back to PD
Marzuk et al. (2018) [13]	82	M	ESI/TI	Antimicrobials	PD continued
Hibi et al. (2017) [15]	89	M	ESI/TI	Removal of catheter	Palliative care
Renaud et al. (2019)[17]	69	F	ESI/TI	Removal of catheter	Switch back to PD
Lo et al. (2013) [19]	56	M	ESI/TI	Removal of catheter	Converted to HD
Renaud et al. (2019) [17]	63	M	ESI/TI	Removal of catheter	Converted to HD
Kameyama et al. (2007) [20]	51	F	ESI/TI	SCRR	Converted to HD
Yoshimura et al. (2018) [12]	56	M	ESI/TI	SCRR	Converted to HD
Inoue et al. (2018) [18]	61	M	TI	Removal of catheter	Switch back to PD
Current case (2026)	58	M	TI	Removal of catheter	Converted to HD

The first case involved an 82-year-old man with ESRD due to DKD. He had concurrent ESI caused by *M. abscessus* and *Staphylococcus hominis*. The patient underwent unroofing, followed by a seven-week course of triple antimicrobial agents consisting of amikacin (AMK), ofloxacin (OFLX), and CAM, which successfully resolved the infection [13]. The second case was a 50-year-old man who also had concurrent ESI. He received an eight-week course of triple antimicrobial agents consisting of ciprofloxacin (CPFX), CAM, and rifampicin (RFP). Due to concerns regarding colony formation, surgical drainage was performed. The combination of debridement and antimicrobial therapy allowed the continuation of PD without interruption [14].

The two previously reported cases both underwent several weeks of triple antimicrobial therapy and avoided PD catheter removal. This case involved monotherapy with LVFX alone. It remains uncertain whether this was appropriate as initial antimicrobial therapy, but removal of the PD catheter two days after the emergence of multidrug resistance likely contributed to the favorable outcome. A review of previous literature indicates that catheter removal was undertaken in nine out of 11 cases, and the official statement jointly issued by the American Thoracic Society (ATS) and the Infectious Diseases Society of America (IDSA) in 2007 also emphasizes that foreign body removal is essential for successful treatment of *M. abscessus* infections [4]. In case of *M. abscessus*-related TI, it is necessary to consider situations where prompt PD catheter removal is critical.

On the other hand, although only two cases have been reported, they demonstrate that PD can be continued without PD catheter removal. Therefore, when selecting a treatment approach, it is important to individualize therapy by considering the background of each case and the antimicrobial susceptibility of the bacterial species.

## Conclusions

This case illustrates the therapeutic challenges of *M. abscessus*-related TI in PD patients. Although prompt PD catheter removal often leads to successful treatment when antimicrobial therapy fails, as seen in this case, establishing the optimal strategy for *M. abscessus*-related TI requires further research and consensus.
